# Rapid 3 s Curing: What Happens in Deep Layers of New Bulk-Fill Composites?

**DOI:** 10.3390/ma14030515

**Published:** 2021-01-21

**Authors:** Danijela Marovic, Matej Par, Ana Crnadak, Andjelina Sekelja, Visnja Negovetic Mandic, Ozren Gamulin, Mario Rakić, Zrinka Tarle

**Affiliations:** 1 Department of Endodontics and Restorative Dentistry, School of Dental Medicine, University of Zagreb, Zagreb 10000, Croatia; marovic@sfzg.hr (D.M.); anacrnadak@gmail.com (A.C.); andelina.sekelja96@gmail.com (A.S.); visnja.mandic1@gmail.com (V.N.M.); tarle@sfzg.hr (Z.T.); 2 Institute for Physics and Biophysics, School of Medicine, University of Zagreb, Zagreb 10000, Croatia; ogamulin@sfzg.hr; 3 Institute of Physics, Zagreb 10000, Croatia; mrakic@ifs.hr

**Keywords:** 3 s curing, bulk-fill composites, flexural strength, flexural modulus, degree of conversion, aging, radiant exitance, addition–fragmentation chain transfer

## Abstract

This study assessed the influence of rapid 3 s light curing on the new generation of bulk-fill resin composites under the simulated aging challenge and depths up to 4 mm. Four bulk-fill materials were tested: two materials designed for rapid curing (Tetric PowerFill—PFILL; Tetric PowerFlow—PFLW) and two regular materials (Filtek One Bulk Fill Restorative—FIL; SDR Plus Bulk Fill Flowable—SDR). Three-point bending (*n* = 10) was used to measure flexural strength (FS) and flexural modulus (FM). In the 3 s group, two 2 mm thick specimens were stacked to obtain 4 mm thickness, while 2 mm-thick specimens were used for ISO group. Specimens were aged for 1, 30, or 30 + 3 days in ethanol. The degree of conversion (DC) up to 4 mm was measured by Raman spectroscopy. There was no difference between curing protocols in FS after 1 day for all materials except PFLW. FM was higher for all materials for ISO curing protocol. Mechanical properties deteriorated by increasing depth (2–4 mm) and aging. ISO curing induced higher DC for PFLW and FIL, while 3 s curing was sufficient for PFILL and SDR. The 3 s curing negatively affected FM of all tested materials, whereas its influence on FS and DC was highly material-specific.

## 1. Introduction

The use of dental bulk-fill composites in restorative dental medicine has been increasing in recent years due to the simplicity of their application in thick 4–5 mm layers and clinical performance similar to conventional composites [1,2]. However, demands for quick solutions have enticed the manufacturers of dental materials to shorten the chairside treatment duration even more. It has been claimed that the curing time can be reduced to only 3 s with the prerequisite high radiant exitance of photopolymerization devices of 3000 mW/cm^2^ or higher [3]. At the same time, the mechanical and curing properties that impact a patient’s health and longevity of restorations should not be compromised.

The exposure reciprocity hypothesis claims that the light exposure time can be reduced if the radiant exitance (mW/cm^2^) is increased, as long as the energy density (J/cm^2^) is the same. However, this concept has been heavily criticized in past studies [4,5,6]. Higher radiant exitance combined with short exposure time commonly resulted in a lower degree of conversion (DC), hardness, flexural strength (FS), and modulus (FM), in contrast to the higher exposure times combined with lower radiant exitance of the curing unit [4,5,6]. Nevertheless, some exceptions are noticed [7,8]. Anomalies in the exposure reciprocity concept are observed for some low-viscosity composites, explained by bimolecular free radical termination. Namely, in the rapid initiation reaction, a large number of photons activate a large number of photoinitiators and free radicals. At such a high concentration, free radicals annihilate each other, thus reducing the possibility of further activation of monomer species and propagation of radical polymerization. The result is a heterogeneous polymer network and lower DC of such materials [9,10]. In high-viscosity materials, the filler particles decelerate this reaction, so the consequences are not as clearly visible as in low-viscosity materials.

Thus, the desired reduction of the curing time requires further modification of the organic matrix. One dental manufacturer has launched two materials, high-viscous Tetric PowerFill (Ivoclar Vivadent, Schaan, Liechtenstein; PFILL) and low-viscous Tetric PowerFlow (Ivoclar Vivadent, Schaan, Liechtenstein; PFLW), with the suggested curing time of 3 s with the radiant exitance of 2700–3300 mW/cm^2^ in the blue-violet spectrum, available with the accompanying curing unit Bluephase PowerCure (Ivoclar Vivadent, Schaan, Liechtenstein) [3,11,12]. Low-viscosity material PFLW has a virtually identical composition as its predecessor, Tetric EvoFlow Bulk Fill. PFILL, on the other hand, has a fundamentally altered polymerization process, made possible by incorporating a unique reagent β-allyl sulfone in the organic matrix [3]. This compound promotes the addition-fragmentation chain transfer (AFCT) reaction, which competes with conventional free radical polymerization, typical for most dental composites [13,14,15,16]. Uncontrolled free-radical polymerization results in long crosslinked polymers of various molecular weights with unreacted monomers trapped within the polymer network. Consequently, resin composites are brittle materials in which free monomers can leach out of the material and cause allergic and toxic reactions in a biological system. AFCT reagent β-allyl sulfone promotes the step-like growth of the polymer chains, arresting the long polymer chain formation while simultaneously creating free radicals that initiate a new propagating radical, leading to new chain formation [14,15]. In the polymer systems with β-allyl sulfone, a mixed polymerization reaction consisted of radical and step-like chain growth occurs [14]. As a result, shorter polymer chains and a more homogeneous structure are generated, with lower glass transition temperature and consequently improved mechanical properties [13]. 

This alteration in material design was apparently sufficient to enable short light exposure times with high radiant exitance without adverse consequences, as reported in a small number of studies that investigated these materials [15,17,18,19,20]. There was no difference in the DC, depth of cure, Vickers microhardness, FS, and FM for PFILL, when polymerized for 10 s with moderate irradiance or 3 s with high irradiance [15]. The only recorded difference was in faster polymerization kinetics for PFILL compared to its predecessor Tetric EvoCeram Bulk Fill with similar composition, but without AFCT reagent [15], and in polymerization rate in comparison to conventional composite [17]. This was evidently reflected in the maximum shrinkage force rates, which were higher and developed faster for PFILL in comparison to conventional curing [18]. However, linear shrinkage, shrinkage force, and DC were equal regardless of the curing protocol for PFILL [18]. Rapid curing with high radiant exitance seems to influence the crosslinking density of the polymer network, where low-viscosity composites showed higher crosslinking density than high-viscosity composites [19]. This fact was later confirmed by Graf and Ilie [16] for high-viscosity material PFILL, who demonstrated higher versatility of crosslinking density throughout the specimens in rapid high-radiant exitance curing in comparison to the ISO curing protocol. This resulted in inferior mechanical properties at the bottom of 2-mm thick specimens.

The gradual decrease in crosslinking density is most likely related to light attenuation. Light attenuation is determined by absorption by photoinitiators and pigments, reflection on filler/resin interface, changes in refractive indices during polymerization and temperature changes, as well as air bubbles incorporated in the structure of composite material. Additionally, the optical path length of through the material is one of the crucial factors that reduce the number of photons available to initiate the polymerization in deeper areas [21]. It is a well-known fact that bulk-fill composites have higher light transmission, which allows their placement in 4 mm thick layers [22], but that does not imply that the surface and the 4 mm depth have identical properties [23,24,25].

Although bulk-fill composites have been used for more than ten years, ISO 4049 standards for testing their mechanical properties remain adjusted for conventional composites limited to 2 mm placement. According to ISO 4049, the specimens for FS and FM measurements should be fully polymerized with recommended light curing from the top and bottom of 2 mm thick specimens to minimize the effect of the curing conditions on the outcomes. Nonetheless, the clinical situation is very different. Dentists place the bulk-fill composites in 4 mm layers and can cure them only from the top surface. With the additional shortening of curing time to only 3 s for certain bulk-fill materials, it is evident that ISO recommended conditions for measurement of FS and FM are not clinically relevant and that the obtained values do not correspond to those achieved in vivo. However, the majority of the scientific community follows the ISO regulations. Owing to them, the reported FS and FM between studies can be compared. Thus, the comparison of ideally polymerized materials according to the ISO protocol and a challenged polymerization protocol closer to clinical conditions could give a valuable information.

The present study aimed to examine the DC, FS, and FM of a new generation of bulk-fill composites at 0–2 mm and 2–4 mm depths cured with ultra-short very high-radiant exitance light and compare them to ISO-recommended curing on 2 mm thick specimens. FS and FM were measured after one day, 30 days, and 30 days followed by three days of artificial aging in ethanol. DC was measured after 30-day exposure to saline solution and three days of drying in the desiccator to ensure completeness of the post-polymerization [26]. The null hypothesis was that the following factors do not influence DC, FS, and FM:

1. curing protocol,
2. curing depth and
3. aging.

## 2. Materials and Methods

### 2.1. Materials

Four bulk-fill composite materials were tested, two of high-viscosity: PFILL and Filtek One Bulk Fill Restorative, 3M ESPE, St. Paul, MN, USA (FIL) and two low-viscosity materials: PFLW and SDR Plus Bulk Fill Flowable, Dentsply Caulk, Milford, MA, USA (SDR). The composition of the materials is given in Table 1. 

### 2.2. Methods

#### 2.2.1. Characterization of the Curing Light

For spectral characterization of Bluephase PowerCure, spectrometer Ocean Optics HR4000 (Ocean Optics, Largo, FL, USA) was used. The fiber optic sensor of the spectrometer was placed in the center of the 9 mm light guide of the curing unit. The recording was taken in the 3s, turbo, and high-power mode.

The camera was used to record the light output with and without the light guide using a neutral density filter (OD = 1) or white paper as a background.

#### 2.2.2. Flexural Strength and Flexural Modulus

Custom-made metal split-molds with 16 × 2 × 2 mm incisions were filled with unpolymerized materials, covered with a transparent polyethylene terephthalate (PET) foil (25 × 10 mm) on top and bottom surface, and pressed with glass plates to extrude the excess material. The specimens were polymerized with Bluephase PowerCure in direct contact to the foil using overlapping irradiations. A silicone key was made to ensure the reproducible positioning of the light guide. Two light-curing protocols were used:

1. ISO group—specimens polymerized according to ISO 4049 [27]—6 × 20 s with 950 mW/cm^2^ (3 times on each side, 6 times in total), *n* = 10/material/time point;
2. 3 s group—double, stacked specimens polymerized 3 × 3 s with 2700 mW/cm^2^ on only one side (3 times in total), *n* = 10/depth/material/time point.

**Figure 1:**
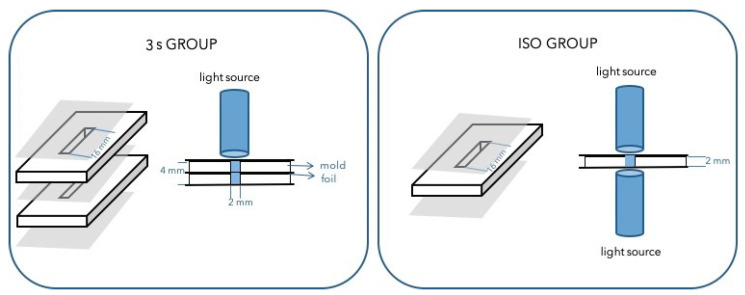
Experimental setup for two polymerization protocols.

The radiant exitance of the curing unit was measured three times every day before use with a Bluephase meter (Ivoclar Vivadent, Schaan, Liechtenstein). The experimental setup is depicted in Figure 1. For the 3 s group, two molds were stacked together so that their openings were aligned entirely, and fixations prevented their movement on two sides. A PET foil separated the molds. Material flashes were removed by polishing with silicon carbide paper (Grit500/P1000, Buehler, Lake Bluff, IL, USA).

Specimens were stored in Eppendorf tubes (10 specimens each) in 4 mL saline solution in the dark, at 37 °C. Three time points were defined following a previous study [28]:

- 1 day (saline),
- 30 days (saline), and
- 33 days (30 days in saline and 3 days in 70% ethanol).

After storage, the specimens were loaded until fracture in a universal testing machine (Ultratester, Ultradent Products Inc., South Jordan, UT, USA) using a three-point bending test device with the span between supports of 12 mm and the crosshead speed of 1 mm/min. Flexural strength was calculated using the formula:

σ = 3FL/2bh^2^ (MPa) (1)

F represents the maximum force; L span between supports; b is the width, and h is the specimen’s height. The flexural modulus of elasticity is calculated according to the formula:

E = FL^3^/4bh^3^d (GPa) (2)

in which d represents the deflection of the specimen according to the load F.

#### 2.2.3. Degree of Conversion

Spectra of polymerized and unpolymerized specimens were taken with an FT-Raman spectrometer (Spectrum GX, PerkinElmer, Waltham, MA, USA) with a NdYaG laser at a wavelength of 1064 nm, with a laser power of 800 mW and a resolution of 4 cm^−1^. Specimens (*n* = 5) that were previously used to test mechanical properties were stored in saline at 37 °C for 30 days, fractured in a three-point bending test, and stored in the dark in a desiccator for the next three days. The spectra of the upper and lower surface of each specimen were recorded. The exposed specimen size was 0.5 mm in diameter. A total of 150 scans were recorded for each spectrum. The spectra were processed in the Matlab program (Mathworks, Natick, MA, USA).

The DC was calculated by comparing the relative change of the integrated band intensities at 1640 cm^−1^ (aliphatic C=C bonds) and the reference band at 1610 cm^−1^ (aromatic C=C bonds) of unpolymerized and polymerized specimens. The DC is calculated by including the values of the integrated intensities in the formula:

DC = 1− R_polymerized_/R_unpolymerized_ (%) (3)

where R = (aliphatic C=C integrated intensity)/(aromatic C=C integrated intensity).

#### 2.2.4. Statistical Analysis

The Shapiro–Wilk test and inspection of normal Q-Q plots showed that the data within individual experimental groups did not deviate significantly from the normal distribution. Therefore, all statistical analyses were performed using parametric tests. For one-way ANOVA, the assumption of homoskedasticity was verified by the Levene test. The Mauchly test verified the assumption of sphericity for comparisons using repeated measurements ANOVA.

The DC values among materials were compared by one-way ANOVA, with Tukey’s post-hoc correction for multiple comparisons. A comparison of DC at different depths (0 mm and 2 mm) for each material was performed by *t*-test for independent measurements assuming homogeneous variances. One-way ANOVA with Tukey’s post-hoc correction was used to compare the DC for each material at more than two depths (0 mm, 2 mm upper, 2 mm lower, 4 mm). The DC measured after ISO polymerization and 3 s polymerization was compared by *t*-test for independent measurements assuming homogeneous variances.

Required sample size was estimated in a preliminary study which showed that *n* = 10 is sufficient for obtaining statistical power over 0.85. The FS and FM values were compared among materials within an individual time point using one-way ANOVA with Tukey’s post-hoc correction. The FS and FM values among different time points for a particular material were compared by ANOVA for repeated measurements with Bonferroni post-hoc correction. For FM and FS, pairwise comparisons (top vs. bottom) were performed by *t*-test for independent measurements and homogeneous variances. For specimens representing different layer thicknesses (top and bottom), for each layer thickness, the FM and FS values were compared separately among the materials for each time point using one-way ANOVA with Tukey correction for multiple comparisons. In the same way, for each layer thickness, the FM and FS values were compared separately among the time points for a particular material using ANOVA for repeated measurements and Bonferroni corrections for multiple comparisons. Pairwise comparisons of FS and FM for specimens polymerized by the ISO protocol and the 3 s polymerization protocol were performed by *t*-test for independent measurements assuming heterogeneous variances.

For all tests, *p*-values less than 0.05 were considered statistically significant. The statistical analysis was performed using SPSS (version 20, IBM, Armonk, NY, USA).

## 3. Results

### 3.1. Characterization of the Curing Light

**Figure 2:**
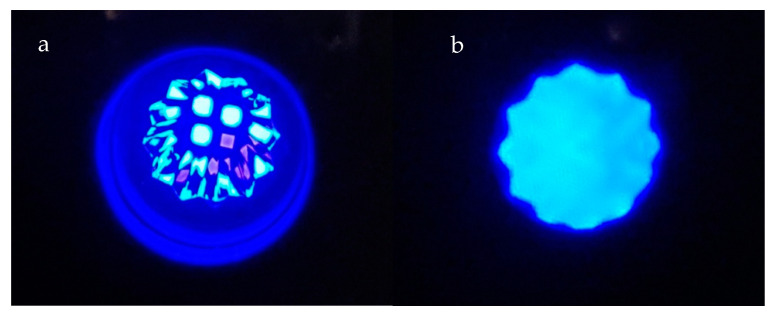
(**a**) Position of blue and violet light-emitting diodes in the Bluephase PowerCure, surrounded by the mirrors for detecting the backlight for determination of the proximity of the tooth structure; (**b**) light output at the tip of the light guide.

**Figure 3:**
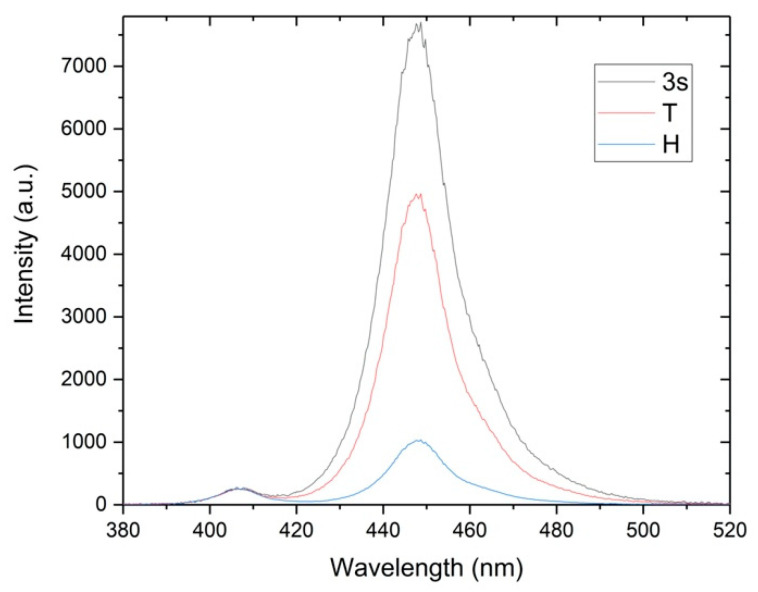
The emission spectrum of the Bluephase PowerCure in the 3s, turbo (T), and high power (H) curing mode.

The optical output of the Bluephase PowerCure curing unit was analyzed. The examined curing unit has four light-emitting diodes, three blue, and one violet (Figure 2a), recorded using a neutral density filter (OD = 1). Figure 2b shows the light output at the tip of the light guide, recorded with camera using white paper as diffusor. The recorded emission spectrum is shown in Figure 3. It can be easily recognized that the total spectrum is a compound of two parts. One smaller part of the spectrum is centered around 404 nm, while the dominant part of the spectrum is centered about 447 nm. The relative radiant exitance of the violet peak at 404 nm remains the same regardless of the curing mode, while the radiant exitance of the 447 nm varies.

### 3.2. Flexural Strength and Flexural Modulus

#### 3.2.1. 3 s vs. ISO

**Figure 4:**
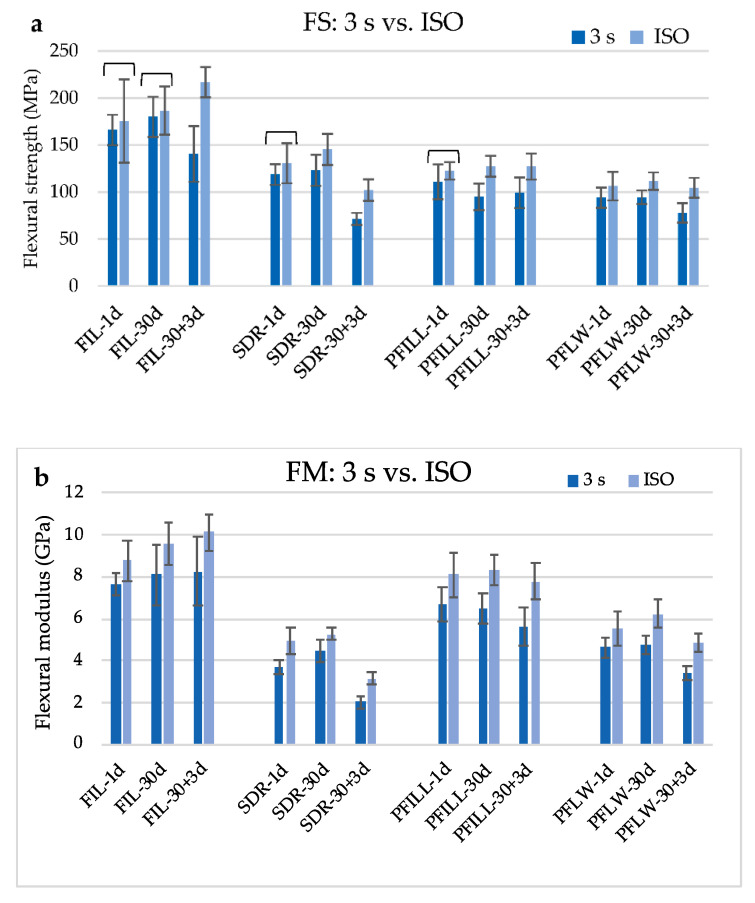
Comparison of (**a**) flexural strength and (**b**) flexural modulus of tested materials polymerized according to the ISO protocol and the top specimens polymerized with 3 s protocol (mean values ± 1 standard deviation). Brackets indicate statistically homogeneous groups for comparison of ISO and 3 s protocol (*p* < 0.05).

Figure 4 shows the comparison of FS and FM between top specimens of the 3 s group and ISO group. FM was significantly higher for all materials and time points when polymerized with ISO protocol. There was no difference in FS between two polymerization protocols after 1 day for all tested materials, except for PFLW. Additionally, there was no difference in FS between polymerization protocols for FIL after 30 days.

#### 3.2.2. 3 s Group—Depth and Aging

For 3 s curing, the curing depth significantly affected the degradation of the mechanical properties. Top specimens had higher FS and FM values than the bottom specimens within the same material and time point. The only exception was SDR after 30 days and PFILL after 1 and 30 days, whose FS of the upper and lower specimens did not show statistically significant differences. The FM in the 3 s group was significantly lower in the bottom specimens than the top for all materials except SDR after 1 day.

**Figure 5:**
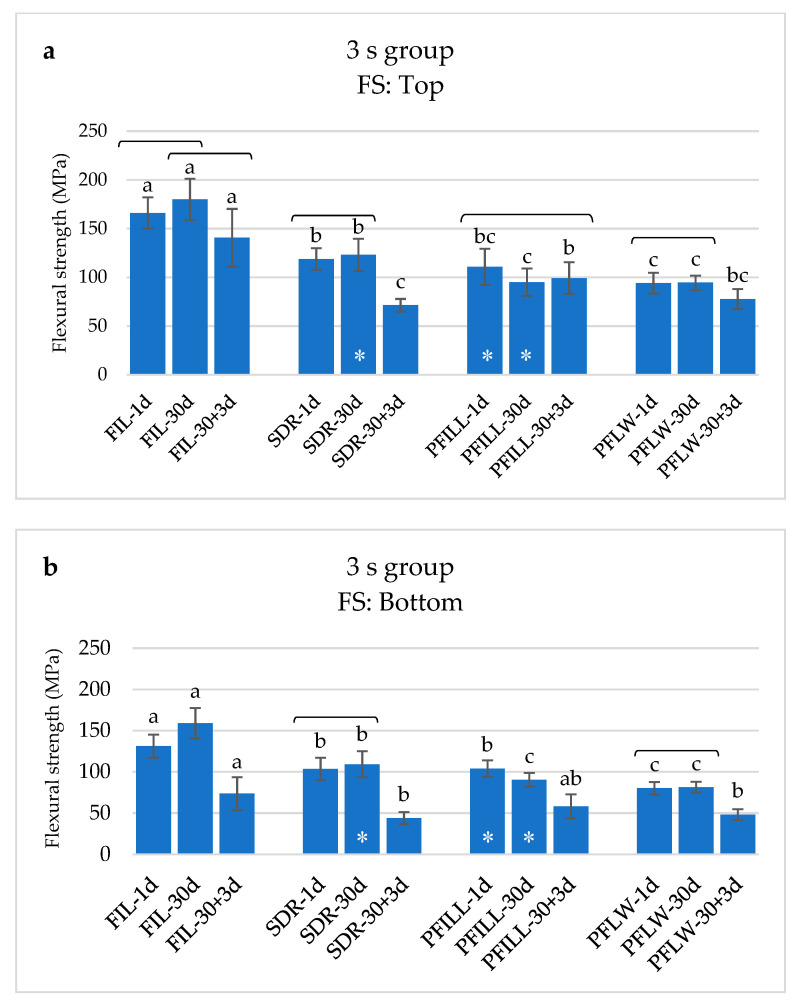
Flexural strength (mean values ± 1 standard deviation) for materials polymerized with 3 s protocol: (**a**) top specimens, (**b**) bottom specimens. The same letters show statistically homogeneous groups for a particular time point. Brackets indicate statistically homogeneous groups for each material. Asterisk denotes no statistically significant differences between top (**a**) and bottom specimens (**b**) of the same material and time point (*p* < 0.05).

Figure 5 shows that FS was the highest for FIL, for all time points, upper and lower specimens. SDR follows it, then PFILL and PFLW. The degradation of FS due to aging was more significant for the bottom than for the top specimens, especially in the 30 + 3-day group. In the 30 + 3-day group, SDR and PFLW showed the lowest values (63.97 ± 10.68 MPa for SDR and 70.28 ± 9.70 MPa for PFLW), below the minimum recommended values of 80 MPa according to ISO standard 4049: 2009.

**Figure 6:**
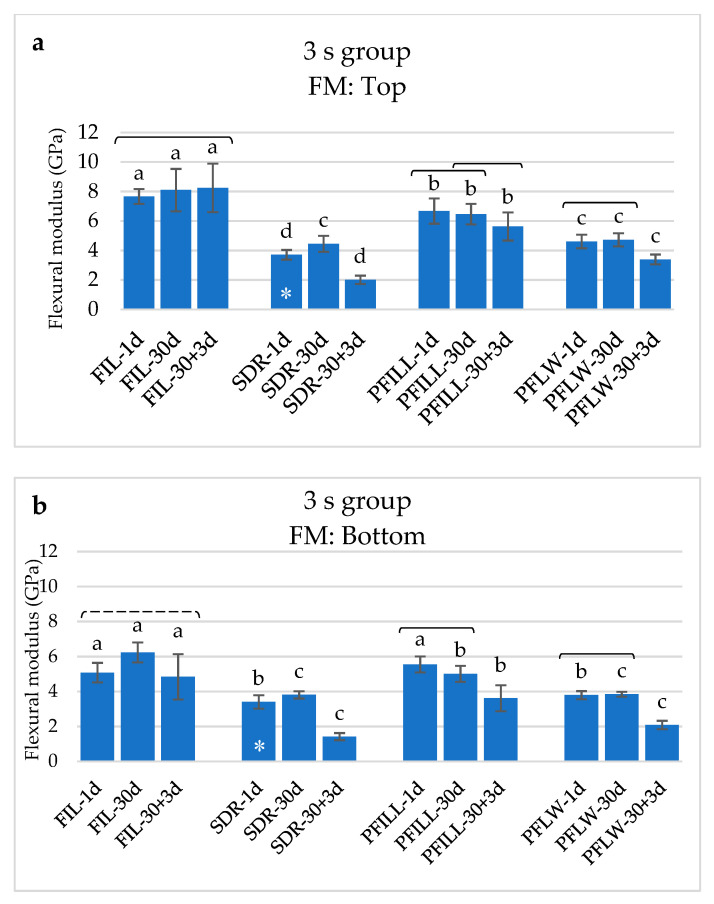
Flexural modulus (mean values ± 1 standard deviation) for materials polymerized with 3 s protocol: (**a**) top specimens, (**b**) bottom specimens. The same letters show statistically homogeneous groups for a particular time point. Brackets indicate statistically homogeneous groups for each material (*p* < 0.05). Dashed brackets indicate statistically homogeneous groups for a particular material, which are not similar to the column in between (in cases where the time dependence is not monotonous), *p* < 0.05. Asterisk denotes no statistically significant differences between top (**a**) and bottom specimens (**b**) of the same material and time point.

The FM (Figure 6) of the top specimens was higher than the FM for bottom specimens of the same material in all types of materials. Again, FIL was the material with the highest FM, followed by PFILL and PFLW, while SDR had the lowest FM under the tested conditions. FIL was also the only material without weakening FM compared to 1 and 30 + 3 days, valid for both the bottom and top specimens.

### 3.3. Degree of Conversion

#### 3.3.1. 3 s vs. ISO

**Figure 7:**
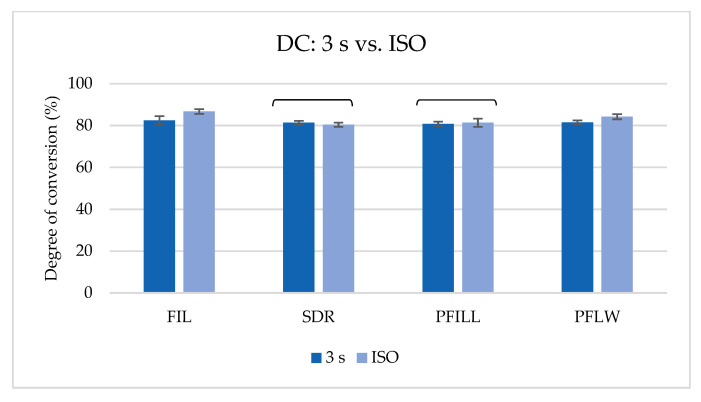
Comparison of degree of conversion (%) between ISO at 2 mm and 3 s group at a 2 mm depth of the top specimen (mean values ± 1 standard deviation). Brackets denote statistically similar pairs of 3 s and ISO groups of the same material (*p* < 0.05).

A comparison of the DC of the top specimens polymerized with ISO or 3 s protocol is shown in Figure 7. All tested materials exhibit a very high DC at a 2 mm depth, over 80%. There was no difference in polymerization 3 s with a very high radiant exitance or ISO polymerization in SDR and PFILL. FIL and PFLW showed higher values when polymerized with ISO curing protocol.

#### 3.3.2. 3 s Group—Curing Depth

**Figure 8:**
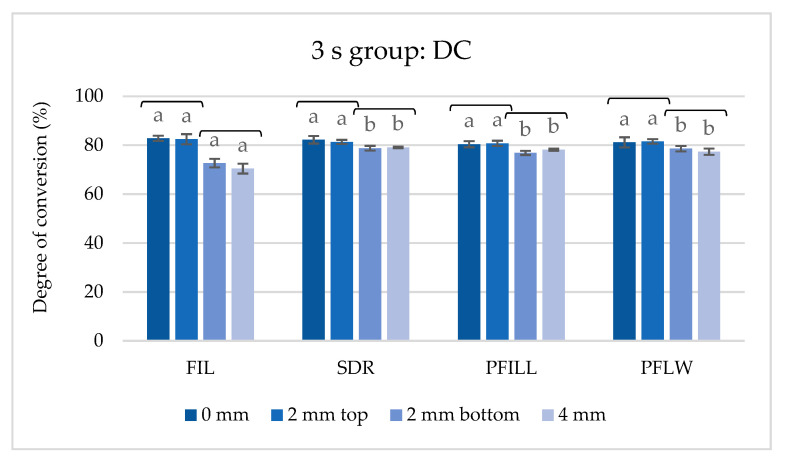
The degree of conversion (%) for materials polymerized with 3 s protocol (mean values ± 1 standard deviation). The same letters indicate statistically homogeneous groups for a single depth, while brackets indicate statistically homogeneous groups for a single material (*p* < 0.05).

Figure 8 shows the results of the 3 s group, polymerized at depths up to 4 mm. All materials showed statistically equal values of the DC on the upper specimen, 0 and 2 mm. At the same time, FIL had the weakest polymerized bottom specimens at 2 and 4 mm, as well as the most considerable difference between the upper and lower specimen. The bottom specimens on the other three tested materials were statistically equal and better polymerized than FIL. All materials at all tested depths showed very high conversion rate values, with a bottom to surface ratio (4 mm to 0 mm) of more than 0.8.

## 4. Discussion

This study is the first to examine the influence of 3 s curing with high radiant exitance in relation to curing depth on the FS, FM, and DC of bulk-fill materials designed explicitly for such polymerization. Tested properties were evaluated after one day, one month after light activation, and after accelerated aging in ethanol. DC, FS, and FM were influenced by light-curing protocol, curing depth, and aging, but not in the same manner for all the materials. Thus, all three null hypotheses were partially rejected.

### 4.1. Curing Protocol

The first null hypothesis was partially rejected: FM was significantly lower in 3 s than in the ISO group, while FS and DC were dependent on the material type. PFILL and SDR had statistically similar DC values regardless of the polymerization mode, while FIL and PFLW confirmed better polymerization by ISO protocol. This result is not surprising for PFILL. As mentioned earlier, PFILL encompasses β-allyl sulfone that promotes the so-called step-polymerization. This reagent terminates radical chain polymerization in one chain, simultaneously forming a sulfonyl radical and a new double carbon bond [13]. The sulfonyl radical is a new source for the initiation of polymerization. Combined with camphorquinone and germanium-based photoinitiator Ivocerin activated by high energy levels, numerous initiation sites seem to contribute to adequate DC in 3 s polymerization protocol. On the other hand, FIL contains an addition-fragmentation monomer (AFM) [29], chemically similar to AFCT reagent β-allyl sulfone in PFILL. However, FIL is evidently better cured with moderate-radiant exitance light-curing with a longer exposure time. This might be caused by high-molecular-weight AUDMA, which most likely limits monomers’ mobility in a rapidly forming network.

PFLW does not contain AFCT, but a high DC is achieved by closely matched refractive indices of the organic matrix and filler in the unpolymerized state [30], making it a highly translucent material with a translucency of about 28%. During polymerization, the refractive index of the polymerized organic matrix in this material changes, and the material becomes less translucent (<10%) [3]. In the 3 s polymerization, the polymerization reaction will be significantly faster than in the ISO group, which will also cause a quicker loss of translucency of PFLW [17]. Higher polymerization rate and faster loss of translucency probably caused slightly higher DC results in PFLW in ISO polymerization. A similar behavior was noted for the predecessor of PFLW, Tetric EvoFlow Bulk Fill, where lower DC was found at 4 mm than at 1 mm depth [31].

FM was higher for ISO polymerization for all materials and test periods. This finding is in agreement with the hypothesis that the moderate radiant exitance with extended curing times generates high DC, densely crosslinked polymer networks, which yield higher modulus [32]. Similar results are found in other studies examining the dependence of FM to different radiation energies [33,34,35]. Radiant exposure, a product of radiant exitance and the exposure time [6], shows the amount of energy per unit of surface area the specimens received. In our study, ISO specimens were illuminated six times with an energy of 19 J/cm^2^, a total of 114 J/cm^2^, while 3 s specimens received only three irradiations of 8.1 J/cm^2^, a total of 24.3 J/cm^2^. Thus, it is understandable that almost five times more received energy applied in a more gradual manner was manifested in a higher FM. It is presumed that high radiant exitance applied over a short exposure time produces high crosslinking density in new generations of bulk-fill composites [16,19]. Nonetheless, a lower amount of energy in the 3 s group than the ISO group is a plausible reason for higher FM in the ISO group. Still, such high energy is clinically never applied, and the absolute FM values achieved by 3 s curing are within the acceptable limits.

Our research suggests no difference in FS concerning the polymerization protocol, except for low-viscosity PFLW. However, it should be emphasized that although there was no difference in the mentioned materials after one day of immersion in water, the differences appeared after prolonged exposure to saline and accelerated aging using ethanol in favor of ISO polymerization. This finding supports the theory that the created polymer network differs significantly when the material is initially illuminated with a high radiant exitance. Contrary to the expectations that improvements in crosslinking density due to a higher radiant exitance would improve the material’s resistance to aging, our results showed that the 3 s specimens were more prone to plasticization and degradation during accelerated aging with ethanol. Our previous work has demonstrated that the response of composite materials to high radiant exitance is highly material-specific [19]. Par et al. studied the influence of the rapid high radiant exitance on the micromechanical properties of bulk-fill composite materials. Both conventional and bulk-fill composites of low-viscosity showed a reduction in microhardness (11–48%) using high radiant exitance. In contrast, there was no change in high-viscosity composite materials regardless of the polymerization method [19]. In addition, FS seem to be less affected by the polymer network’s crosslinking density than FM [34].

In the current study, materials that did not show a difference in DC irrespective of the illumination protocol, PFILL and SDR, retained the same FS in the one-day group. FIL’s seemingly contradictory behavior, with lower DC when polymerized by the 3 s protocol on one side and the resistance to aging-induced weakening of the FS with 3 s polymerization (1 and 30 days) on the other, can be explained by the mentioned high viscosity. FIL was the material with the highest filler vol% in the study. Moreover, it must be noted that even though FIL had statistically lower DC with 3 s polymerization, the absolute DC values for this polymerization protocol at 2 mm of the top specimen were around 80%, i.e., statistically similar to those for PFILL, SDR, and PFLW. The comparison of mechanical properties in 3 s vs. ISO polymerization was also performed on the (better polymerized) top specimens between the 3 s and ISO groups.

All materials were almost equally polymerized, thus the differences in FS between materials can be ascribed to filler characteristics, such as size, shape, volume, and silanization. It is clear that the material with the lowest filler volume, PFLW with 46%, was also the material with the lowest FS, while FIL had the highest filler volume and the highest FS.

### 4.2. Curing Depth

Bucuta and Ilie [36] reported that bulk-fill composite materials transmit light better than conventional composites. Despite higher translucency, the deeper layers do not achieve optimal polymerization as the surface due to light attenuation [23,25]. This assumption was also confirmed in our study in which the top specimens were statistically more polymerized than the bottom ones for all tested bulk-fill materials. To test the mechanical properties of bulk-fill materials in deeper layers, especially in challenging polymerization conditions of only three seconds, we developed experimental settings with separable top and bottom specimens that were polymerized only from top to more realistically mimic the clinical situation. 

The results show that increasing the depth generally reduces the FS and FM of each material, thus rejecting the second null hypothesis. The exception is PFILL after 1 and 30 days, and SDR after 30 days, which showed statistically similar FS on 0–2 mm as well as 2–4 mm. Both SDR and PFILL are characterized by high translucency in the unpolymerized state [3,36] which could explain high DC values at 4 mm, with less than 3% difference in absolute DC values between 4 mm and surface. 

On the other hand, FIL and PFLW were slightly less polymerized, reducing the DC for 12% from the surface to 4 mm in the case of FIL. High opacity of FIL did not hinder its depth of cure when polymerized with moderate radiant exitance for 20 s [37]. In our study, rapid high radiant exitance light activation probably caused a reduced number of initiation sites in deeper layers 2–4 mm from the light source due to light scattering. Similar behavior of PFLW could be ascribed to the previously mentioned fast onset of opacity and consequent decline in DC with increasing depth. Correspondingly, lower DC could explain a further drop in the FS and FM.

The influence of the PET foil, which separated the specimens, deserves further investigation. In preliminary tests with a UV-vis spectrometer, the selected foil had the highest light transmission in the wavelength range from 350 to 550 nm. However, comparing the DC values at the bottom of the upper specimen (at 2 mm) and the surface of the lower specimen (∼2 mm) between which the foil was positioned, a statistically significant difference was found for all materials in the 3 s polymerization protocol. This means that foil influence was not negligible and that it could emphasize the differences in the mechanical properties between the upper and lower specimens. The polymerization process in a particular layer (depth) depends not only on the number of photons reaching that particular layer but also on the presence of an already ongoing polymerization process in the upper layers, which progresses deeper [38]. The foil prevented the transition of free radicals from the top to the bottom specimen, which probably inhibited polymerization. Furthermore, this effect could have negatively impacted Ivocerin-containing materials, which have maximum absorption in the violet spectrum. As seen in Figure 3, the violet LED has low radiant exitance. Combined with the poor penetration of the violet wavelengths, it is possible that this photoinitiation could not be appropriately activated in the bottom specimen 2–4 mm from the light source [39]. This is also the biggest drawback of this study. As these assumptions are tested, a study of the behavior of bulk-fill materials polymerized for 3 s on extracted teeth is planned for future research.

On the other hand, our study was designed to allow statistical comparison of mechanical properties between the 3 s group and the ISO group. Despite the formulae for calculating flexural strength and elastic modulus do contain parameters that are supposed to formally account for different specimen geometries, there are abundant empirical data showing that a simple mathematical correction that uses factoring with geometry parameters (describing specimen cross-section) raised to first/second/third power cannot fully compensate for variations in specimen geometry [40]. The failure mechanics of brittle inhomogeneous materials is complex due to probabilistic effects related to the presence of structural flaws within the material; both empirical observations and theoretical modeling indicate that flexural strength decreases with increasing specimen size [41]. This effect depends on the characteristics of individual structural flaws within a specimen and cannot be fully compensated for by simple calculations of flexural strength and elastic modulus according to ISO 4049.

This experimental setup also allows for a distinction between the upper, better-polymerized part of the specimen in contrast to the bottom specimens with lower DC. In a recent innovative study by El-Askary et al. [42], the 4 mm thick specimens for a three-point bending test were light-cured only from the top surface without the foil. However, the results obtained in that study were confusing since the obviously less polymerized specimens cured with an 8 mm distance from the light source obtained higher FS than the same material polymerized with only 2 mm distance from the light source.

This seemingly contradictory result was explained by the polymer network’s heterogeneity. Light attenuation through the material likely caused higher cross-linking at the specimen surface, while longer polymer chains with less cross-linking formed in the lower parts. It was hypothesized that the linear polymer structure in the bottom part of the specimen was more flexible [34,43,44]. It is suspected to permit higher deformation before brittle failure [45], leading to artificially increased FS. Unfortunately, the degree of cross-linking, DC, and FM were not measured in that study [42], so the conclusions were not unequivocal. However, our study design supported their assumptions as we found lower FM and DC in the bottom specimens. Even though the foil separating the specimens led to the mentioned limitations of the present study, the advantage of this study design was the differentiation of the behavior at different depths of the bulk-fill composites.

Unlike other studies employing the same curing light with radiant exitance at 3000 mW/cm^2^ or more [15,16,17,18,19], our study employed radiant exitance of 2700 mW/cm^2^, which is on the lower end of the recommended range by the manufacturer of the PFILL and PFLW [11,12]. Knowing that the radiant exitance of curing lights decreases over time [46,47], it is of clinical interest to know what the repercussions of this alteration are.

### 4.3. Aging

The decrease in mechanical properties of the material as a consequence of aging can be caused by the degradation of any structural component of the composite material, such as resin matrix, filler particles, and the interface between filler and matrix [28]. Ideally, resin composites should be chemically stable, and their mechanical properties should not show significant deterioration during aging. However, the conditions to which dental restorative materials are exposed are demanding: constant humidity, strong repeated masticatory forces, enzymatic degradation, temperature changes, and many others. It has been stated that the FS of conventional composites decreases by about 60% after one year of exposure to water [48]. In order to simulate aging in the laboratory, water exposure, the thermocycling process, and also simple alcohol exposure is often used. The use of ethanol-based solutions or other organic solvents has been associated with the degradation of mechanical properties [49]. In this study, the effect of aging in water after 30 days and additional accelerated aging after a further three days in ethanol was studied. Ethanol was chosen because of its similar solubility with dimethacrylate monomers used in most composite materials. Alcohol molecules easily penetrate the material and cause widening of the intermolecular spaces, disrupting the physical bonds between polymer chains. However, it does not affect the chemical bonds in crosslinked polymer networks. Poorly crosslinked materials are prone to resin plasticization [50] and degradation of mechanical properties [28] upon exposure to ethanol. 

Accordingly, prolonged exposure to water and alcohol impaired the quality of the material in the present study. The third null hypothesis is also rejected. In general, the weakening of mechanical properties was more pronounced in the lower specimens, which were less polymerized. The FS of PFILL on the top specimens was stable even after alcohol exposure, while the decrease with aging was significant for the bottom specimens, with no significant difference between the upper and lower specimens after 1 and 30 days. The same behavior is shown by FIL, with the difference that the lower specimens had significantly lower FS, which can be related to the lower DC achieved in the lower specimens. On the other hand, low-viscosity materials SDR and PFLW showed good stability of FS in water, but by exposure to alcohol, it dropped significantly to values below 80 MPa, which is the lower limit for composite materials set according to ISO standard 4049 [27].

The increase of FM in the bottom specimens of FIL could be the consequence of the under-polymerization with the 3 s curing protocol. Lower photon count in the lower parts of the bottom specimen could lead to linear polymer development, which offers less rigidity [43]. This has led to lower FM after one day of storage. The post-polymerization [26] in the following month was the most likely cause of the increase in FM. In the 30 + 3-day group, the exposure to ethanol has again reduced the FM to the statistically similar values as those obtained at 1 day. This 30-day increase was also evident in the FS in the bottom specimen of FIL, but the decrease after exposure to ethanol was below the 1-day FS values, with a statistically significant difference.

## 5. Conclusions

Within the conditions of the current study, rapid high radiant exitance curing led to reductions of the FM of tested bulk-fill composites. Furthermore, all the tested mechanical properties and the DC were lower in deep areas 2–4 mm away from the light source. Aging and exposure to ethanol led to a material-dependent deterioration of FS and FM, which was more pronounced in deeper areas. The composite material designed for rapid curing with high radiant exitance containing AFCT reagent initially showed equal FS and DC as with ISO curing, but its FM was negatively affected. 

## Figures and Tables

**Table 1:** Composition of tested materials provided by the manufacturers.

Viscosity	Material (Manufacturer, Abbreviation)	Resin	Filler wt%/vol%	Photoinitiators
**High-** **viscosity**	Filtek One Bulk Fill Restorative, 3M ESPE, St. Paul, MN, USA (FIL)	AUDMA, DDDMA, proprietary AFM	76.5/58.5	CQ/amine
	Tetric PowerFill, Ivoclar Vivadent, Schaan, Liechtenstein (PFILL)	Bis-GMA, Bis-EMA, UDMA, PBPA, DCP, β-allyl sulfone	76/53	CQ/amine, Ivocerin
**Low-** **viscosity**	SDR Plus Bulk Fill Flowable, Dentsply Caulk; Milford, CT, USA (SDR)	Modified UDMA, TEGDMA, dimethacrylate and trimethacrylate resins	70.5/47.4	CQ/amine
	Tetric PowerFlow, Ivoclar Vivadent, Schaan, Liechtenstein (PFLW)	Bis-GMA, Bis-EMA, UDMA, DCP	68/46	CQ/amine, Ivocerin

Bis-GMA: bisphenol A-diglycidyl dimethacrylate, UDMA: urethane dimethacrylate; Bis-EMA: ethoxylated bisphenol A dimethacrylate; DCP: tricyclodecane-dimethanol dimethacrylate; PBPA: propoxylated bisphenol A dimethacrylate; CQ: camphorquinone; AUDMA: aromatic urethane dimethacrylate; DDDMA: 1, 12-dodecanediol dimethacrylate; AFM: addition-fragmentation monomer; TEGDMA: triethylene glycol dimethacrylate.

## Data Availability

The datasets generated and analyzed during the current study are available from the corresponding author on reasonable request.
